# Contemporary estimates of the risk of end-stage renal disease in the first decade of proliferative lupus nephritis

**DOI:** 10.1186/ar3946

**Published:** 2012-09-27

**Authors:** MM Ward, M Tektonidou

**Affiliations:** 1 National Institute of Arthritis and Musculoskeletal and Skin Diseases, National Institutes of Health, Bethesda, MD, USA; 2 School of Medicine, National University of Athens, Greece

## Background

End-stage renal disease (ESRD) is a major cause of morbidity and costs in patients with systemic lupus erythematosus (SLE). Patients with proliferative lupus nephritis are at greatest risk of ESRD, but with recent treatment advances, the proportion of patients with proliferative nephritis who develop ESRD may be decreasing. We sought to learn the risk of ESRD in patients with proliferative lupus nephritis enrolled in studies since 1990.

## Methods

In a systematic literature review, we searched PubMed, Embase, and the Cochrane Database of Systematic Reviews from inception to November 2011 to identify published articles on the risk of ESRD in lupus nephritis. We also searched references of retrieved articles and reviews. We excluded articles with fewer than 10 patients, less than 1 year of follow-up, those primarily of children, and those that did not report ESRD (dialysis or renal transplantation) as a specific outcome. Of 1,144 unique articles from the searches, we did a full-text review of 373 articles. One hundred and fifty-five articles met inclusion criteria, reported relevant data and were not duplicate reports on the same cohort. Thirty-one studies began enrollment in 1990 or later. Here we examined the 14 studies (15 arms) that reported outcomes of patients with proliferative lupus nephritis. We computed weighted averages of the proportion with ESRD at the mean follow-up. For studies with more than one treatment arm, we pooled estimates across arms.

## Results

The 15 arms were from one prospective observational study, five retrospective observational studies, and nine clinical trials. Samples ranged from 9 to 117 patients. All but one study were done at referral centers, and nine specifically excluded patients with elevated serum creatinine at baseline. Mean serum creatinine at entry was 1.2 mg/dl and mean proteinuria was 3.0 g/day. Mean duration of lupus nephritis at entry was 1.1 years. Mean follow-up was 4.5 years. At 1 to 3 years of lupus nephritis, the pooled estimate of ESRD was 4.5% (based on five arms); at 4 to 6 years, the pooled estimate was 6.9% (three arms); at 7 to 9 years, the pooled estimate was also 6.9% (five arms); and at 10 to 11 years, the pooled estimate of ESRD was 3.9% (two arms).

## Conclusion

In patients with proliferative lupus nephritis enrolled in studies since 1990, the risk of ESRD over the first decade of lupus nephritis is under 7%. This estimate is largely based on long-term follow-up of clinical trials and studies that excluded patients with renal insufficiency at baseline.

